# Allogeneic Platelet-Rich Gel Supernatant Reprograms the Cytokine and Growth Factor Microenvironment in an Equine In Vitro Inflammatory Tendon System

**DOI:** 10.3390/ijms27094006

**Published:** 2026-04-29

**Authors:** Jorge U. Carmona, Catalina López

**Affiliations:** 1 Grupo de Investigación Terapia Regenerativa, Departamento de Salud Animal, Universidad de Caldas, Calle 65 No 26-10, Manizales 170004, Colombia; 2 Grupo de Investigación Patología Clínica Veterinaria, Departamento de Salud Animal, Universidad de Caldas, Calle 65 No 26-10, Manizales 170004, Colombia; catalina.lopez@ucaldas.edu.co

**Keywords:** platelet-rich plasma, platelet-rich gel, tendinopathy, cytokines, growth factors, hyaluronic acid, systems biology, horse, regenerative medicine

## Abstract

Tendinopathy involves a dysregulated inflammatory microenvironment in which cytokines, growth factors (GF) and extracellular matrix components interact dynamically. Platelet-rich plasma (PRP) is widely used as a regenerative therapy, but its mechanisms of action in inflamed tendon remain unclear. This study evaluated whether platelet-rich gel supernatant (PRGS) reprograms the inflammatory–anabolic mediator network in an equine in vitro tendon explant system stimulated with lipopolysaccharide (LPS). Tendon explants were cultured under six experimental conditions, including non-stimulated control, LPS-stimulated control, and LPS combined with 25% or 50% PRGS or platelet-poor gel supernatant (PPGS). Cytokines, GF, and hyaluronic acid (HA) were quantified at 1 h and 48 h and analyzed using linear mixed-effects models, mediator ratios, correlation networks, and principal component analysis. PRGS contained higher concentrations of PDGF-BB (2044 pg/mL, 95% CI 1382–2706) and IL-1ra (1196 pg/mL, 95% CI 424–1967) compared with PPGS. In LPS-stimulated explants, PRGS significantly increased IL-1ra and PDGF-BB, while IL-1β and HA exhibited significant time-dependent changes (F = 8.675 and F = 10.752, respectively). The PDGF-BB:HA ratio remained consistently higher in PRGS-treated groups (F = 46.100, *p* < 0.001). Multivariate analysis showed that the first two principal components explained 62% of the total variance and revealed coordinated shifts in mediator organization over time. These findings indicate that PRGS does not simply suppress inflammation but actively reprograms the tendon microenvironment toward a regulatory and reparative phenotype within this experimental system.

## 1. Introduction

Tendinopathy is a prevalent musculoskeletal disorder affecting both equine athletes and humans [1,2,3,4]. It is characterized by extracellular matrix (ECM) disorganization, altered tenocyte phenotype, and a persistent dysregulated inflammatory microenvironment [5,6]. In horses, superficial digital flexor tendon (SDFT) injury represents a naturally occurring overuse tendinopathy with clinical, biomechanical, and histopathological features closely resembling human Achilles tendinopathy [1,2,3,4]. Because of its comparable size, hierarchical collagen organization, mechanical loading patterns, and spontaneous degenerative lesions, the equine tendon is increasingly recognized as a valuable translational model for investigating tendon biology and regenerative strategies relevant to human medicine [1,2,3,4].

The current understanding of tendinopathy has evolved from a purely degenerative paradigm toward a model in which inflammation and innate immune activation play central roles in tendon pathology. Early concepts emphasized the apparent absence of classical inflammatory infiltrates and therefore characterized tendinopathy primarily as a degenerative condition (“tendinosis”). However, advances in molecular and cellular biology have demonstrated the presence of immune cells, inflammatory mediators, and cytokine signaling pathways within diseased tendon tissue, supporting the concept that inflammatory mechanisms actively contribute to tendon degeneration and failed tissue repair [7,8].

Tenocytes express pattern recognition receptors, including toll-like receptor 4 (TLR4), enabling them to detect endogenous damage-associated molecular patterns and exogenous pathogen-associated signals [9]. Activation of TLR4 signaling promotes nuclear factor kappa B (NF-κB)–dependent transcription of pro-inflammatory mediators such as interleukin-1β (IL-1β) and tumor necrosis factor-α (TNF-α), which contribute to extracellular matrix degradation and disruption of tendon homeostasis. Conversely, regulatory cytokines including interleukin-4 (IL-4) and interleukin-1 receptor antagonist (IL-1ra) counterbalance IL-1β–driven inflammatory signaling and promote resolution of inflammation and restoration of tissue equilibrium [7,8,10].

In experimental systems, lipopolysaccharide (LPS), a well-characterized TLR4 agonist, is widely used to model innate immune activation and inflammatory responses in tendon tissue [11]. Accordingly, the present study focuses on early inflammatory responses occurring within the acute phase of tendon injury, as represented by the 48 h experimental time frame, consistent with previous evidence in tendon and musculoskeletal inflammation [11,12].

Growth factors (GF) are equally critical in tendon repair and remodeling. Platelet-derived growth factor-BB (PDGF-BB) stimulates tenocyte proliferation and ECM synthesis, while transforming growth factor beta-1 (TGF-β1) regulates collagen production, cellular differentiation, and ECM organization [13,14]. Hyaluronic acid (HA), beyond its structural function, participates in inflammatory signaling and ECM homeostasis [15,16]. Therefore, the simultaneous assessment of pro-inflammatory cytokines, regulatory cytokines, and anabolic GF is essential. This approach allows us to determine whether a therapeutic intervention suppresses isolated mediators or reprograms the tendon microenvironment toward a reparative phenotype.

Platelet-rich plasma (PRP)–related hemocomponents have gained considerable interest in regenerative medicine for tendinopathy [17,18,19]. PRP is a plasma-based formulation containing platelets with variable concentrations of blood cells (WBCs), depending on the preparation protocol. In addition to platelet-derived mediators, PRP includes soluble plasma proteins and coagulation components that contribute to its biological activity [20,21].

Upon activation, PRP polymerizes into platelet-rich gel (PRG), a three-dimensional fibrin matrix that entraps platelets and residual cells. This fibrin scaffold functions not only as structural support but also as a dynamic regulator of mediator release, enabling the gradual liberation of GF and cytokines. Furthermore, the fibrin network acts as a provisional ECM that may facilitate cell trafficking, adhesion, proliferation, and differentiation, thereby contributing to tissue repair beyond soluble signaling alone [22,23]. During clot retraction, PRG releases a soluble supernatant enriched in bioactive mediators derived from platelets, WBC, and plasma. Platelet-rich gel supernatant (PRGS) therefore represents the cell-free soluble fraction generated from activated PRP and reflects the signaling component of this regenerative strategy [24,25].

In a previous mechanistic in vitro study using normal equine tendon explants, we demonstrated that PRGS modulated inflammatory and anabolic mediator release in a concentration-dependent manner, with a 25% concentration inducing a more favorable profile characterized by reduced IL-1β and increased IL-4 and IL-1ra [26]. However, that system evaluated healthy tendon tissue without an inflammatory stimulus, limiting its translational relevance to active tendinitis.

Beyond isolated mediator quantification, tendinopathy can be viewed as a dynamic network disorder [7,27]. In this system, cytokines, GF, and ECM components interact in a temporally regulated and highly interconnected manner. Traditional univariate approaches may fail to capture these interdependencies. From a systems biology perspective, biological responses to regenerative therapies should be interpreted as coordinated network shifts rather than independent molecular changes [28,29].

Accordingly, the objective of this study was to evaluate the effects of PRGS, compared with platelet-poor gel supernatant (PPGS), on the cytokine and GF microenvironment of an equine in vitro tendinitis system induced by LPS challenge. We first characterized the temporal behavior of individual mediators using mixed-model univariate analyses to assess concentration- and time-dependent effects on IL-1β, TNF-α, IL-4, IL-1ra, PDGF-BB, TGF-β1, and HA. Subsequently, we applied multivariate analytical approaches to determine whether PRGS induced coordinated shifts in the inflammatory–anabolic mediator network.

We hypothesized that PRGS would not merely attenuate individual pro-inflammatory cytokines but would reprogram the cytokine and GF network, shifting the tendon microenvironment from a defined LPS-induced pro-inflammatory state toward a regulatory and reparative phenotype.

## 2. Results

### 2.1. Cellular and Molecular Characterization of Hemocomponents

Platelet and WBC concentrations in whole blood and derived hemocomponents are summarized in Table 1. PRP showed higher platelet concentrations compared to whole blood, whereas PPP exhibited reduced platelet levels. WBC concentrations were lower in PRP than in whole blood and were minimal in PPP. All pairwise comparisons among hemocomponents were statistically significant following Holm adjustment (*p* < 0.05).

Mediator concentrations measured in activated supernatants also differed between PRGS and PPGS (Table 2). PRGS contained significantly higher concentrations of PDGF-BB, TGF-β1, and IL-1ra compared with PPGS (Holm-adjusted *p* < 0.05). TNF-α concentrations were modestly but significantly higher in PRGS, whereas IL-4 showed no statistically significant difference between fractions. Hyaluronic acid concentrations did not differ between PRGS and PPGS.

### 2.2. Effects of PRGS and PPGS on Individual Mediators in the LPS-Induced Tendon Model

#### 2.2.1. TGF-β1

**Figure 1:**
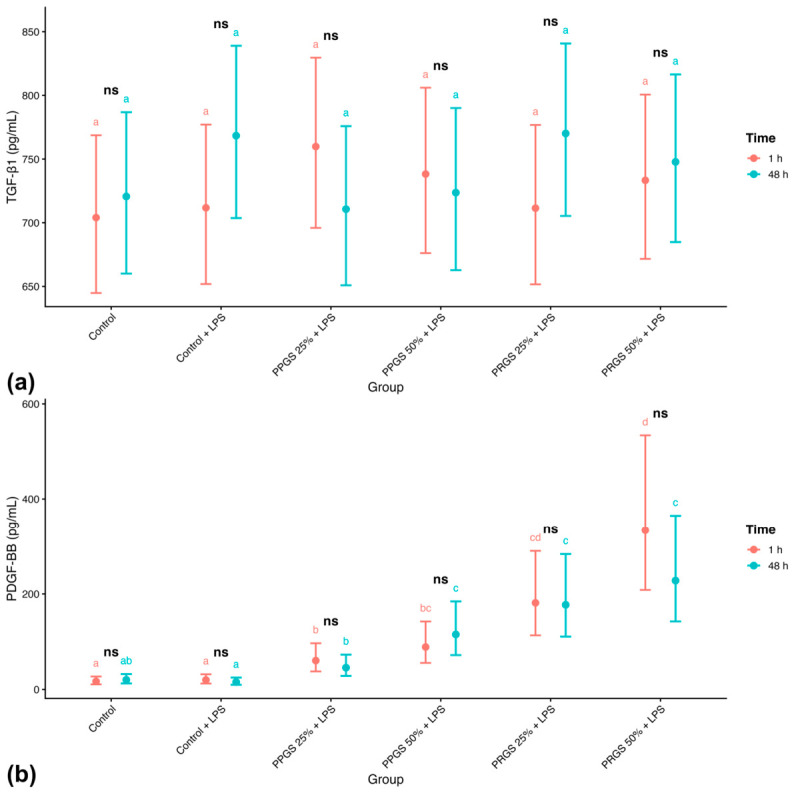
Effects of PRGS and PPGS on growth factor concentrations in the LPS-induced tendon explant system. (**a**) TGF-β1 (pg/mL) and (**b**) PDGF-BB (pg/mL). Points represent estimated marginal means (±95% CI) at 1 h and 48 h. The control group corresponds to LPS-stimulated explants without hemocomponent treatment. Different lowercase letters (a–d) indicate significant differences between treatment groups within the same time point; groups that do not share a letter differ significantly (Holm-adjusted *p* < 0.05), whereas groups sharing at least one letter are not significantly different. “ns” indicates no significant difference between time points within the same treatment group. Abbreviations as in Table 2.

TGF-β1 concentrations did not differ significantly among treatment groups at either 1 h or 48 h (Table 3; Figure 1a). Linear mixed-effects modeling revealed no significant main effect of Group, Time, or Group × Time interaction. Across conditions, values were similar across both control conditions at both time points, and neither PRGS nor PPGS treatments produced detectable changes relative to these references. Back-transformed estimated marginal means (EMMs) showed overlapping 95% confidence intervals across all conditions, and no Holm-adjusted pairwise contrasts reached statistical significance. Temporal comparisons within each group likewise showed no significant changes between 1 h and 48 h.

#### 2.2.2. PDGF-BB

PDGF-BB concentrations were significantly influenced by treatment group (Table 3; Figure 1b), whereas no significant effect of time or Group × Time interaction was detected. Back-transformed EMMs showed consistently higher PDGF-BB concentrations in PRGS-treated explants compared with Control + LPS and both PPGS groups at both time points. Values were similar across both control conditions, whereas PRGS-treated explants showed higher concentrations relative to these references, and PPGS-treated groups remained comparable to Control + LPS. Compact letter displays confirmed significant between-group differences within each time point (Holm-adjusted *p* < 0.05).

#### 2.2.3. TNF-α

**Figure 2:**
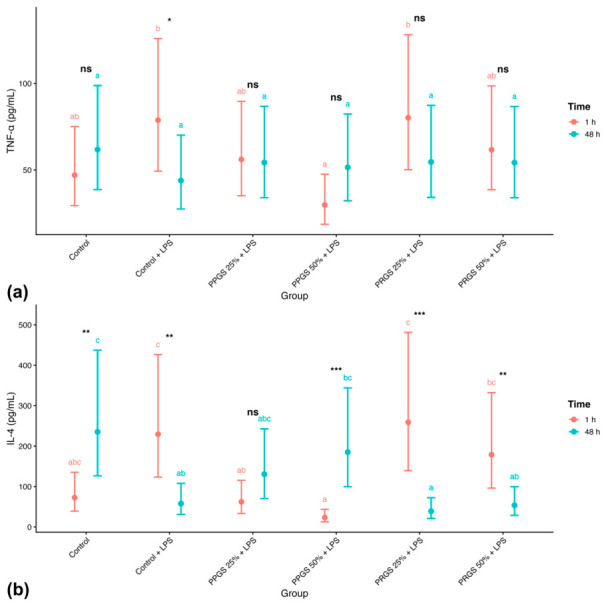
Effects of PRGS and PPGS on inflammatory cytokine concentrations in the LPS-induced tendon explant system. (**a**) TNF-α (pg/mL) and (**b**) IL-4 (pg/mL). Points represent estimated marginal means (±95% CI) at 1 h and 48 h. The control group corresponds to LPS-stimulated explants without hemocomponent treatment. Different lowercase letters (a–c) indicate significant differences between treatment groups within the same time point; groups that do not share a letter differ significantly (Holm-adjusted *p* < 0.05), whereas groups sharing at least one letter are not significantly different. Asterisks indicate significant differences between time points within the same treatment group (* *p* < 0.05, ** *p* < 0.01, *** *p* < 0.001), whereas “ns” indicates no significant temporal difference within that group. Abbreviations as in Table 2.

TNF-α concentrations did not exhibit a significant main effect of Group or Time (Table 3; Figure 2a). A trend toward a Group × Time interaction was observed, but post hoc comparisons did not reveal consistent differences among treatments within time points after Holm adjustment. Values were similar across both control conditions at both time points, and neither PRGS nor PPGS treatments produced consistent differences relative to these references. Back-transformed EMMs showed modest variability across groups; however, 95% confidence intervals largely overlapped. Temporal comparisons within each group were not statistically significant.

#### 2.2.4. IL-4

IL-4 concentrations demonstrated a significant Group × Time interaction (Table 3; Figure 2b), indicating that treatment effects differed between 1 h and 48 h. Within-time comparisons revealed differences among specific treatment groups at one or both time points (Holm-adjusted *p* < 0.05), as reflected by distinct superscript letters. Back-transformed EMMs indicated that PRGS and PPGS treatments produced time-dependent IL-4 responses. Values differed between both control conditions, and PRGS and PPGS treatments showed temporal changes relative to these references. Time-within-group contrasts confirmed significant temporal changes in selected treatment conditions, consistent with the interaction effect.

#### 2.2.5. IL-1β

**Figure 3:**
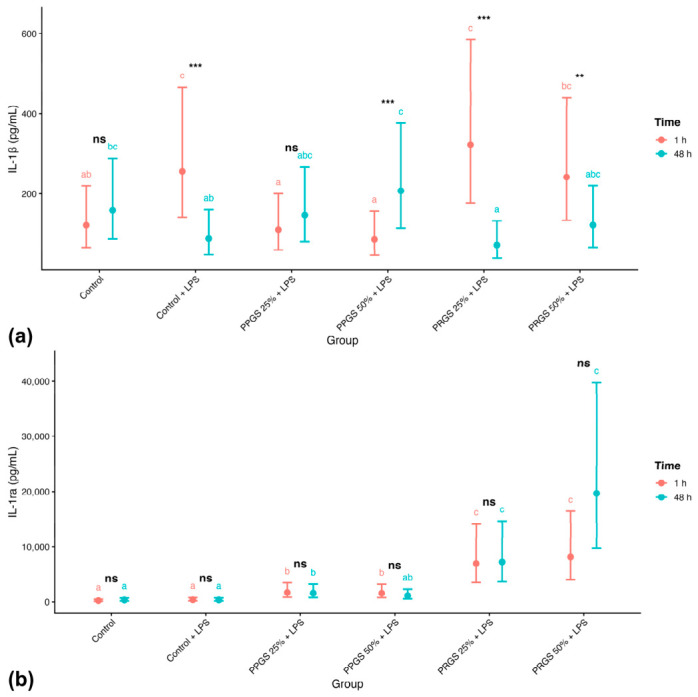
Effects of PRGS and PPGS on pro- and anti-inflammatory mediator concentrations in the LPS-induced tendon explant system. (**a**) IL-1β (pg/mL) and (**b**) IL-1ra (pg/mL). Points represent estimated marginal means (±95% CI) at 1 h and 48 h. The control group corresponds to LPS-stimulated explants without hemocomponent treatment. Different lowercase letters (a–c) indicate significant differences between treatment groups within the same time point; groups that do not share a letter differ significantly (Holm-adjusted *p* < 0.05), whereas groups sharing at least one letter are not significantly different. Asterisks indicate significant differences between time points within the same treatment group (** *p* < 0.01, *** *p* < 0.001), whereas “ns” indicates no significant temporal difference within that group. Abbreviations as in Table 2.

IL-1β concentrations were significantly influenced by time and by the Group × Time interaction (Table 3; Figure 3a). At 1 h, PRGS-treated explants exhibited higher concentrations compared with several other treatment groups, whereas PPGS conditions showed comparatively lower values. Concentrations differed between both control conditions, and PRGS-treated explants showed higher values at 1 h followed by a reduction at 48 h relative to these references. At 48 h, IL-1β concentrations decreased in several groups, particularly under PRGS 25% and Control + LPS conditions. Compact letter displays confirmed differences among treatment groups within time points (Holm-adjusted *p* < 0.05). Temporal contrasts revealed reductions from 1 h to 48 h in Control + LPS, PPGS 50% + LPS, and both PRGS groups, while Control and PPGS 25% + LPS did not exhibit significant temporal changes.

#### 2.2.6. IL-1ra

L-1ra concentrations were significantly affected by treatment group (Table 3; Figure 3b), with no significant main effect of Time or Group × Time interaction. Back-transformed EMMs showed markedly higher concentrations in PRGS-treated explants compared with Control + LPS and PPGS groups at both time points (Holm-adjusted *p* < 0.05). Values were similar across both control conditions, whereas PRGS-treated explants showed higher levels relative to these references, and PPGS groups remained comparable to Control + LPS. Confidence intervals showed minimal overlap between PRGS and non-PRGS conditions, confirming robust treatment-related differences. Temporal comparisons within each group were not statistically significant.

#### 2.2.7. HA

**Figure 4:**
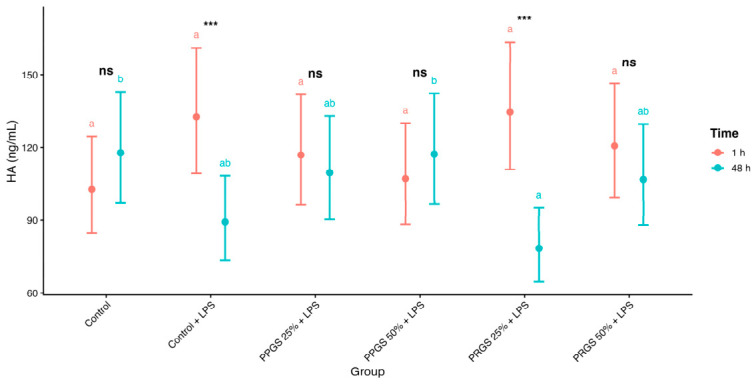
Effects of PRGS and PPGS on hyaluronic acid concentrations in the LPS-induced tendon explant system. Points represent estimated marginal means (±95% CI) at 1 h and 48 h. The control group corresponds to LPS-stimulated explants without hemocomponent treatment. Different lowercase letters (a and b) indicate significant differences between treatment groups within the same time point; groups that do not share a letter differ significantly (Holm-adjusted *p* < 0.05), whereas groups sharing at least one letter are not significantly different. Asterisks indicate significant differences between time points within the same treatment group (*** *p* < 0.001), whereas “ns” indicates no significant temporal difference within that group. Abbreviations as in Table 2.

Hyaluronic acid concentrations were significantly influenced by Time and by the Group × Time interaction (Table 3; Figure 4). Back-transformed EMMs demonstrated changes between 1 h and 48 h that differed across treatment groups. Values differed between both control conditions, and PRGS- and PPGS-treated explants showed similar temporal patterns relative to these references, with limited differences between groups at individual time points. Within-time comparisons revealed minimal between-group differences, whereas temporal contrasts within groups identified significant changes in specific treatments (Holm-adjusted *p* < 0.05).

### 2.3. Correlation Structure of the Mediator Network

**Figure 5:**
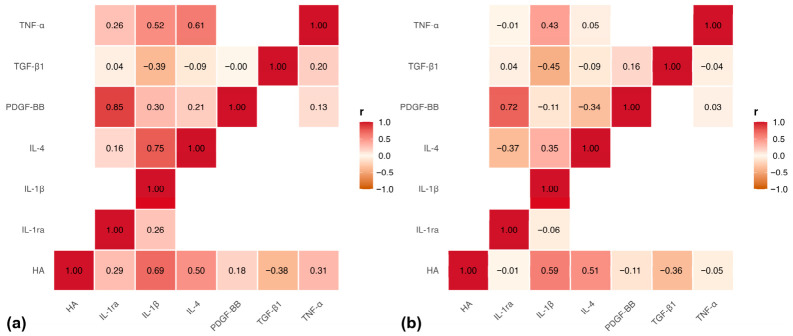
Correlation structure of the mediator network in the LPS-induced tendon explant system. (**a**) Pearson correlation matrix at 1 h and (**b**) Pearson correlation matrix at 48 h. Correlations were computed using log10-transformed mediator concentrations pooled across experimental groups at each time point. Only the lower triangular portion of the matrix is shown. The color scale represents Pearson’s correlation coefficient (r), ranging from −1 (negative correlation) to +1 (positive correlation). Interpretation focused on moderate-to-strong associations (|r| ≥ 0.60) that remained statistically significant after false discovery rate (FDR) adjustment.

At 1 h, several strong associations among mediators were observed (Figure 5a). IL-1β exhibited strong positive correlations with IL-4 and HA. TNF-α also correlated strongly with HA, and PDGF-BB showed a significant positive association with HA.

At 48 h, changes in the correlation structure were observed (Figure 5b). The IL-1β–HA association persisted, whereas some correlations present at 1 h were reduced. PDGF-BB maintained or increased its association with HA at this time point.

### 2.4. Inflammatory—Anabolic Ratios and Microenvironmental Balance

**Figure 6:**
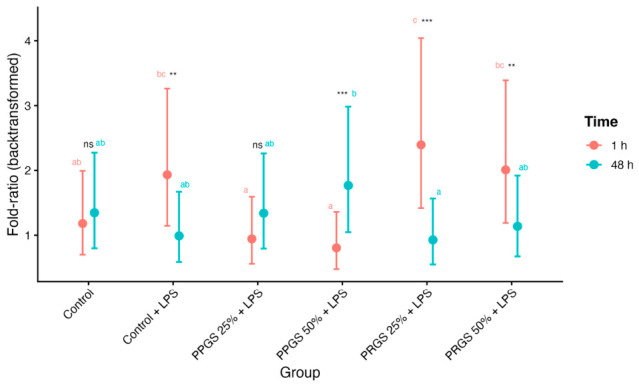
IL-1β:HA fold-ratio at 1 h and 48 h. Back-transformed fold-ratios derived from log10-transformed values are shown for each experimental group at 1 h and 48 h. Points represent estimated marginal means (±95% CI). The control group corresponds to LPS-stimulated explants without hemocomponent treatment. Different lowercase letters (a–c) indicate significant differences between treatment groups within the same time point; groups that do not share a letter differ significantly (Holm-adjusted *p* < 0.05), whereas groups sharing at least one letter are not significantly different. Asterisks indicate significant differences between time points within the same treatment group (** *p* < 0.01; *** *p* < 0.001), whereas “ns” indicates no significant temporal difference within that group. Abbreviations as in Table 2.

The IL-1β:HA ratio showed a significant Group × Time interaction (F = 10.499, *p* < 0.001; Table 4). As illustrated in Figure 6, PRGS-treated explants displayed higher fold-ratios at 1 h compared with 48 h, with significant temporal differences observed in PRGS 25% + LPS and PRGS 50% + LPS. Values differed between both control conditions, and PRGS-treated explants showed higher fold-ratios at 1 h relative to these references, whereas values at 48 h were reduced. Within-time comparisons revealed differences among groups at 1 h, whereas differences between groups were less pronounced at 48 h.

**Figure 7:**
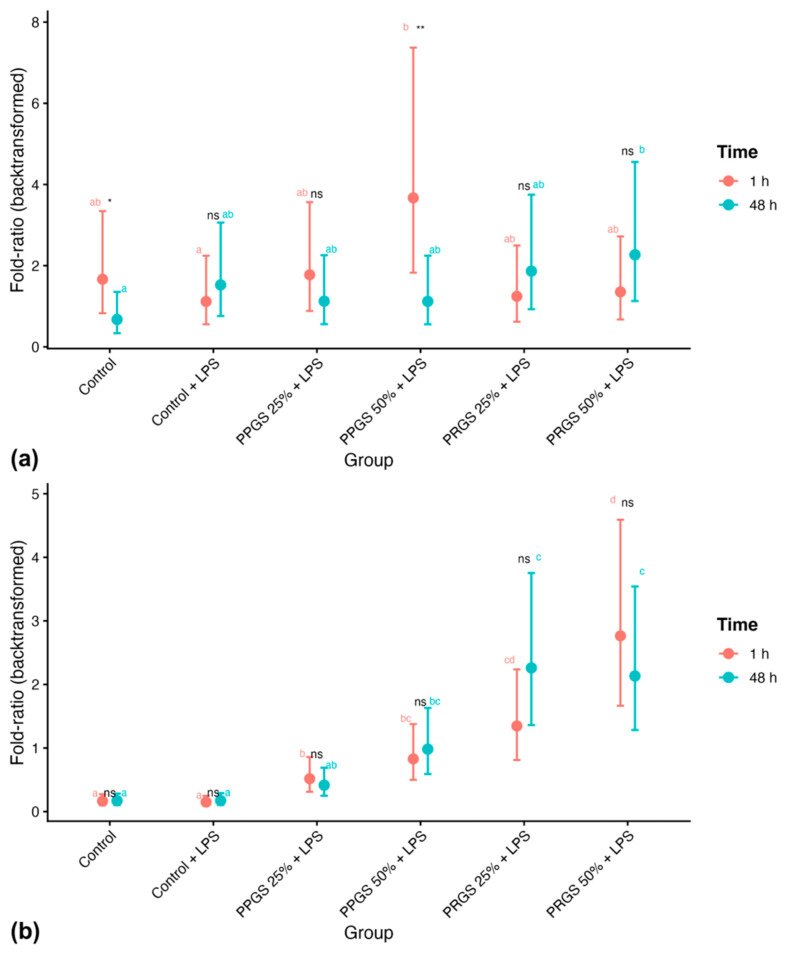
Inflammatory and trophic–matrix ratios at 1 h and 48 h. Back-transformed fold-ratios derived from log10-transformed values are shown for (**a**) IL-1β:IL-4 and (**b**) PDGF-BB:HA across experimental groups at 1 h and 48 h. Points represent estimated marginal means (±95% CI). The control group corresponds to LPS-stimulated explants without hemocomponent treatment. Different lowercase letters (a–d) indicate significant differences between treatment groups within the same time point; groups that do not share a letter differ significantly (Holm-adjusted *p* < 0.05), whereas groups sharing at least one letter are not significantly different. Asterisks indicate significant differences between time points within the same treatment group (* *p* < 0.05; ** *p* < 0.01), whereas “ns” indicates no significant temporal difference within that group. Abbreviations as in Table 2.

The IL-1β:IL-4 ratio also demonstrated a significant interaction effect (F = 3.651, *p* = 0.006; Table 4). As shown in Figure 7a, PRGS 25% + LPS exhibited higher fold-ratios at 1 h relative to 48 h, whereas the remaining groups did not show significant temporal differences. Values differed between both control conditions, and PRGS 25% + LPS showed higher fold-ratios at 1 h relative to these references. Group comparisons within each time point revealed differences at 1 h, particularly among specific treatment groups.

In contrast, the PDGF-BB:HA ratio showed a significant main effect of Group (F = 46.100, *p* < 0.001) without significant effects of Time or Group × Time interaction (Table 4). As presented in Figure 7b, PRGS groups consistently exhibited higher fold-ratios compared with controls at both time points. Values differed between both control conditions, and PRGS groups showed higher fold-ratios relative to these references, whereas PPGS groups remained comparable to Control + LPS. No significant temporal differences were detected within individual groups.

### 2.5. Multivariate Organization of the Mediator Network (PCA and Clustering)

**Figure 8:**
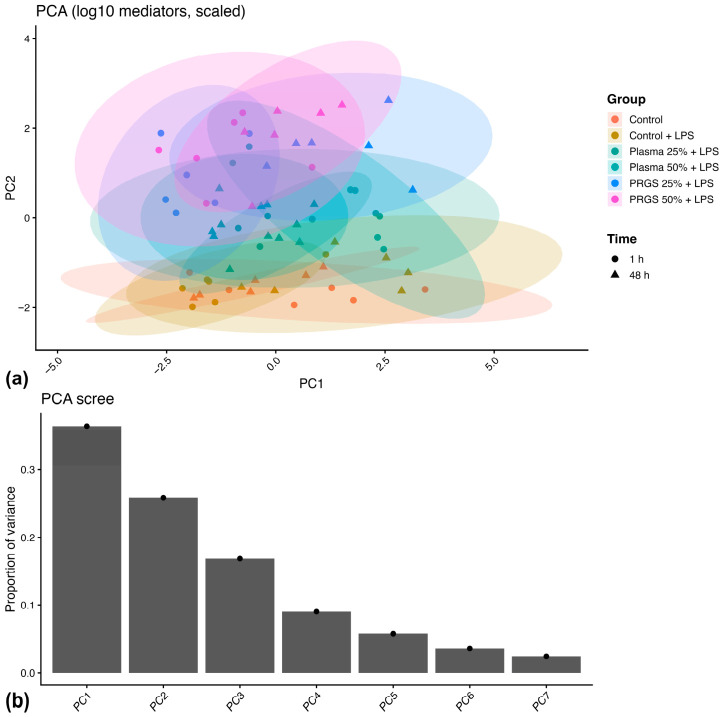
Principal component analysis of mediator concentrations. (**a**) Projection of samples onto the PC1–PC2 space showing the multivariate distribution of mediator profiles across experimental groups and time points. Ellipses illustrate the dispersion of each treatment group in the multivariate space. (**b**) Scree plot showing the proportion of variance explained by each principal component. Gray bars represent the explained variance, and black dots indicate the exact values. PC1 and PC2 accounted for the largest share of the total variance (36.5% and 25.6%, respectively), together explaining approximately 62% of the dataset variability. Subsequent components contributed progressively less variance.

Principal component analysis (PCA) was applied to evaluate the multivariate structure of the mediator data. Projection of samples onto the PC1–PC2 space showed variation in their distribution across experimental groups and time points (Figure 8a). The distribution of samples differed between both control conditions, and treatment groups showed distinct positions relative to these references. The first two principal components accounted for a substantial proportion of the total variance, with PC1 explaining 36.5% and PC2 explaining 25.6%, together representing approximately 62% of the variability in the dataset (Figure 8b).

**Figure 9:**
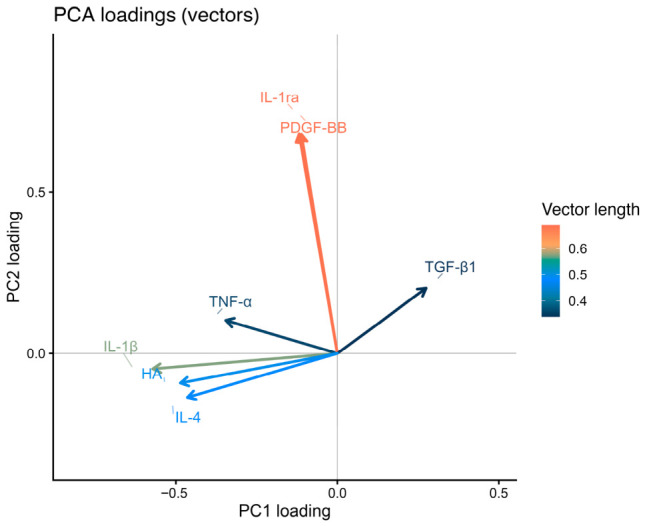
Contributions of mediators to the principal components. PCA loading vectors showing the contribution of each mediator to the first two principal components. Arrow direction indicates the orientation of each variable in the PC1–PC2 space, and arrow length reflects the magnitude of the loading. Colors represent vector length, indicating the relative contribution of each mediator to the multivariate structure. Abbreviations as in Table 2.

Mediator loadings indicated differential contributions to the principal components (Figure 9). PC1 was characterized by positive loadings of TGF-β1 and negative loadings of TNF-α, IL-4, HA, and IL-1β. PC2 was characterized by strong positive loadings of IL-1ra and PDGF-BB.

**Figure 10:**
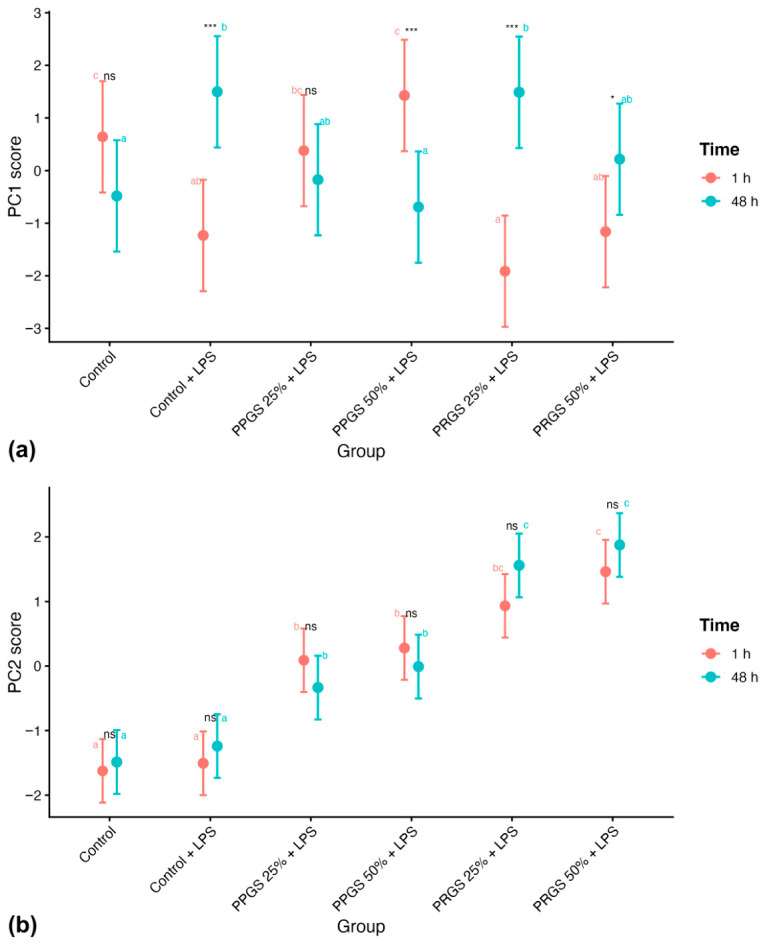
Effects of treatment group and time on principal component scores derived from mediator profiles. (**a**) PC1 scores and (**b**) PC2 scores are shown as estimated marginal means (±95% CI) for each treatment group at 1 h and 48 h. The control group corresponds to LPS-stimulated explants without hemocomponent treatment. Different lowercase letters (a–c) indicate significant differences between treatment groups within the same time point; groups that do not share a letter differ significantly (Holm-adjusted *p* < 0.05), whereas groups sharing at least one letter are not significantly different. Asterisks indicate significant differences between time points within the same treatment group (* *p* < 0.05; *** *p* < 0.001), whereas “ns” indicates no significant temporal difference within that group. No significant temporal differences were detected within treatment groups for PC2 scores.

Analysis of PC scores across groups and time points showed differences between the first two components (Table 5). For PC1, a significant effect of time (F = 6.79, *p* = 0.012) and a Group × Time interaction (F = 14.60, *p* < 0.001) were detected, whereas the main effect of group was not significant (F = 1.03, *p* = 0.406). Estimated marginal means showed variation in PC1 scores across groups and time points (Figure 10a). PC1 scores differed between both control conditions, and treatment groups showed time-dependent changes relative to these references.

For PC2, a significant main effect of group was detected (F = 62.54, *p* < 0.001), whereas neither time (F = 0.81, *p* = 0.373) nor the Group × Time interaction (F = 1.49, *p* = 0.209) was statistically significant (Table 5). Estimated marginal means showed differences among treatment groups at both time points (Figure 10b). PC2 scores were similar across both control conditions, and differences among treatment groups were maintained relative to these references at both time points.

Bootstrap resampling showed stable loading estimates across mediators (Table 6). The variance explained by PC1 remained consistent across bootstrap iterations, and leave-one-donor-out analysis produced similar loading patterns and variance estimates after sequential exclusion of individual donors.

## 3. Discussion

The present study evaluated the effects of PRGS on the cytokine and GF microenvironment of an equine tendon explant system subjected to LPS-induced inflammatory stimulation. The results showed that PRGS was associated with differential patterns of variation across mediators rather than uniform effects. Specifically, PDGF-BB and IL-1ra concentrations differed consistently across treatment groups, whereas IL-1β and HA exhibited time-dependent changes between 1 h and 48 h. Multivariate analysis further indicated variation in mediator profiles across groups and time points, as reflected by differences in principal component scores. Overall, PRGS-treated explants exhibited distinct mediator profiles relative to both conditions [27,28,29]. indicating that PRGS reprograms the mediator network rather than simply suppressing inflammation.

The inclusion of both non-stimulated and LPS-stimulated control conditions allowed differentiation between baseline mediator concentrations and the inflammatory reference state. Across mediators, treatment-related differences were observed relative to one or both reference conditions, depending on the variable and time point.

The LPS-stimulated explant system reproduced several key features of innate immune activation described in tendon inflammation [7,8,10]. LPS acts as a TLR4 agonist that activates NF-κB–dependent transcription of pro-inflammatory cytokines such as IL-1β and TNF-α and promotes ECM remodeling responses in tendon tissue [9]. In the present study, early correlations among IL-1β, TNF-α, IL-4, and HA at 1 h indicated coordinated interactions between inflammatory mediators and ECM components, supporting dynamic interactions during early stages of tissue injury. The strong association between cytokines and HA further suggests that HA may function as a regulatory element linking inflammatory signaling and ECM dynamics within the tendon microenvironment [15,16]. The absence of differences in HA concentrations between PRGS and PPGS suggests that HA levels in the activated supernatants were not determined exclusively by platelet-related mechanisms. Accordingly, HA did not clearly discriminate between both hemoderivatives in this system. Together, these findings indicate that PRGS effects followed two main patterns: (1) consistent differences across treatment groups for selected mediators and (2) time-dependent variation for others, with additional structure revealed by multivariate analysis. This distinction provides a framework for interpreting the biological effects of PRGS within the inflammatory microenvironment.

PRGS treatment resulted in markedly higher concentrations of IL-1ra and PDGF-BB compared with PPGS and control conditions. IL-1ra is a natural competitive antagonist of IL-1 signaling and plays a critical role in limiting IL-1β–mediated inflammatory cascades [30,31]. This is consistent with the higher IL-1ra concentrations observed in PRGS-treated explants. Increased IL-1ra availability may therefore attenuate IL-1β biological activity without completely abolishing inflammatory signaling, thereby preserving physiological inflammatory processes necessary for tissue repair. At the same time, elevated PDGF-BB concentrations indicate strong trophic signaling, as PDGF-BB stimulates tenocyte proliferation, ECM synthesis, and early phases of tendon healing [14,32]. Together, these findings suggest that PRGS introduces both regulatory cytokines and anabolic GF that shift the inflammatory balance toward a reparative microenvironment [21]. These observations are consistent with the primary patterns described above, in which PRGS effects differed across mediators rather than producing uniform responses.

Interestingly, PRGS-treated explants exhibited relatively elevated IL-1β concentrations during the early incubation phase, followed by a reduction at 48 h. Rather than representing a detrimental effect, this transient increase may reflect physiological inflammatory priming. Controlled early inflammatory signaling is increasingly recognized as an essential component of regenerative responses, facilitating immune activation and ECM remodeling before the onset of resolution mechanisms [33,34]. In this context, the simultaneous increase in IL-1ra observed in PRGS-treated explants may represent an intrinsic regulatory mechanism that limits excessive IL-1 signaling while allowing a transient inflammatory phase compatible with tissue repair. This temporal behavior is consistent with the time-dependent variation identified for selected mediators.

In addition, IL-4 warrants specific consideration because it represents an important regulatory cytokine within musculoskeletal inflammatory environments [35,36]. In the present explant system, IL-4 exhibited a significant group × time interaction, indicating that PRGS influenced the temporal dynamics of this cytokine rather than producing a simple uniform increase. Interestingly, IL-4 concentrations did not differ significantly between PRGS and PPGS supernatants, suggesting that the IL-4 patterns observed in the explant cultures primarily reflect tissue-level regulatory responses to inflammatory stimulation rather than passive transfer from the hemocomponent itself. This finding further supports that PRGS effects were not homogeneous across mediators. However, the absence of a direct PRGS-derived source of IL-4 indicates that this cytokine is part of the tissue’s endogenous regulatory response rather than a primary driver of PRGS-mediated reprogramming.

From a mechanistic perspective, IL-4 may contribute to the resolution phase of tendon inflammation by counterbalancing pro-inflammatory signaling pathways and promoting regulatory cellular programs within the tendon microenvironment [37]. This interpretation is consistent with the concept that PRP-related hemocomponents may induce a controlled inflammatory reprogramming in which early inflammatory signals are followed by activation of endogenous regulatory pathways.

Although two PRGS concentrations were evaluated in the present study, the experimental design was not specifically intended to establish a full dose–response relationship. Nevertheless, the mediator patterns observed here are consistent with previous studies suggesting a non-linear response to PRP-related hemocomponents. Interestingly, our findings may also support the concept of a non-linear dose–response relationship for PRP-related hemocomponents. Previous in vitro studies from our group demonstrated that PRGS exerts concentration-dependent effects on joint tissues, where a 25% concentration produced a more favorable anti-inflammatory and anabolic profile than a 50% concentration, whereas higher concentrations were associated with downregulation of ECM anabolic genes and less favorable histological parameters [38]. Mechanistic analyses from that work further showed that PRGS significantly reduced NF-κB gene expression in cartilage explants challenged with LPS, suggesting that platelet-derived mediators may interfere with central inflammatory signaling pathways rather than simply neutralizing individual cytokines. Because NF-κB represents a master regulator of inflammatory gene transcription (including catabolic mediators such as MMP-13 and ADAMTS-4), modulation of this pathway may help explain the coordinated anti-catabolic effects observed in PRG-treated tissues [39].

Similar regulatory patterns have also been described in equine suspensory ligament explant models, where PRGS treatments consistently reduced NF-κB gene expression in LPS-stimulated tissues. [40]. In this context, the markedly elevated IL-1ra concentrations observed in the present study may reflect a compensatory regulatory response triggered by strong inflammatory signaling under higher mediator exposure. Collectively, these observations challenge the traditional assumption that increasing platelet concentrations necessarily produce superior biological effects and instead support the concept of an optimal therapeutic window for PRP-derived signaling molecules [41]. These dose-related considerations align with the heterogeneous patterns observed across mediators in the present study.

Thus, unlike complete cytokine blockade, PRGS appears to modulate inflammatory signaling in a more physiological manner, preserving an early inflammatory phase while providing regulatory mediators that favor resolution [42,43,44]. Overall, these findings are consistent with a non-uniform and context-dependent modulation of the inflammatory microenvironment [45,46].

Analysis of mediator ratios provided additional insight into the inflammatory–anabolic balance of the tendon microenvironment. Ratios such as IL-1β:HA and IL-1β:IL-4 exhibited significant temporal and treatment-dependent variations, reflecting shifts in the relative dominance of inflammatory versus regulatory pathways [47,48]. In contrast, the PDGF-BB:HA ratio remained consistently higher in PRGS-treated groups, suggesting a sustained trophic influence on ECM-related processes. These composite indices may therefore provide integrative indicators of tissue microenvironmental balance, capturing functional relationships between mediators that cannot be readily inferred from individual biomarker concentrations [27,28,29]. The sustained elevation of the PDGF-BB:HA ratio identifies this parameter as a stable signature of PRGS treatment.

Multivariate analyses further supported the concept of coordinated mediator reorganization. PCA revealed two major axes describing the mediator network. The first component was primarily associated with inflammatory and ECM-related mediators, whereas the second component was mainly driven by IL-1ra and PDGF-BB. The strong treatment effect observed for the second component indicates that PRGS predominantly influences regulatory and trophic signaling pathways. Importantly, bootstrap resampling confirmed the stability of this multivariate structure, suggesting that the observed mediator organization was robust and not driven by individual donors [49,50]. This structure indicates that PRGS effects are distributed across multiple mediator pathways rather than concentrated in a single response.

From a biological perspective, the first principal component (PC1) appeared to capture the inflammatory–matrix axis of the tendon microenvironment. The negative loadings of IL-1β, TNF-α, IL-4, and HA suggest that this component reflects coordinated variation among inflammatory cytokines and ECM-associated mediators, which together characterize early inflammatory activation and matrix turnover. In contrast, the second principal component (PC2) was primarily driven by IL-1ra and PDGF-BB, indicating that this axis may represent regulatory and trophic signaling pathways associated with inflammatory resolution and tissue repair. Thus, the separation of these two components supports the interpretation that PRGS influences the balance between inflammatory–matrix responses and regulatory–anabolic signaling within the tendon microenvironment. The significant Group × Time interaction for PC1 combined with the stable Group effect for PC2 demonstrates that PRGS simultaneously engages two independent biological programs: one that evolves over time (inflammation resolution) and another that provides sustained trophic support.

Temporal analysis of the mediator correlation network revealed progressive reorganization of mediator interactions between 1 h and 48 h. Although these correlations do not establish causal relationships, they provide insight into coordinated mediator behavior and highlight potential regulatory modules within the inflammatory microenvironment. The early network displayed high connectivity among cytokines and ECM-associated mediators, whereas the later network exhibited reduced inflammatory coupling and greater modularization. This shift may reflect a transition from an early inflammatory state toward a more regulated microenvironment dominated by trophic and ECM-related signaling. Notably, the persistent association between PDGF-BB and HA at 48 h suggests that trophic signaling becomes increasingly integrated with ECM remodeling during later phases of the response. HA detected in the culture medium may originate from both ECM turnover and de novo synthesis by tendon cells. However, the association between HA and PDGF-BB observed in PRGS-treated explants suggests that HA release may also reflect active-matrix remodeling and fibroblast-related anabolic responses rather than solely matrix degradation. This interpretation is consistent with previous explant studies in equine connective tissues, in which PRG-derived biomaterials modulated HA production and gene expression patterns indicative of anabolic responses in LPS-stimulated synovial membrane and ligament tissues, supporting the concept that platelet-derived mediators can promote ECM remodeling and tissue-repair signaling in inflamed musculoskeletal environments [40,51]. Overall, these observations support a progressive reorganization of mediator interactions over time, consistent with PRGS-mediated modulation of the tendon microenvironment.

Beyond these mechanistic insights, an additional aspect of translational relevance concerns the identification of the most biologically favorable hemoderivative and concentration. In the present study, PRGS-derived treatments consistently promoted a mediator profile characterized by coordinated increases in regulatory cytokines and trophic factors, suggesting that PRGS exert a balanced modulatory effect on the inflammatory microenvironment. Previous work evaluating PRGS effects in equine tendon explants cultured under non-inflammatory conditions demonstrated that this hemoderivative modulates cytokine and GF release in healthy tendon tissue, indicating intrinsic immunomodulatory properties of PRGS [26].

The present study extends those observations by demonstrating that similar modulatory effects occur even in the presence of an LPS-driven inflammatory stimulus, which more closely resembles the innate immune activation observed in diseased tendon tissue. Despite this more challenging inflammatory context, PRGS-based treatments continued to promote a regulatory cytokine environment and coordinated mediator interactions. Together with previous explant studies in equine tendon and suspensory ligament tissues showing that 25% leukocyte-reduced PRGS induces a favorable anti-inflammatory and anabolic mediator profile [26,40], these findings suggest that moderate concentrations of PRGS may provide a biologically balanced stimulus capable of supporting early inflammatory regulation while preserving trophic signaling relevant for ECM remodeling.

Several limitations should be considered. First, the study used an in vitro explant system that cannot fully reproduce the complex cellular, vascular, and mechanical environment of living tendon tissue [52,53]. Second, the inflammatory model relied on LPS stimulation, which represents an acute innate immune stimulus and may not fully recapitulate the multifactorial and chronic processes underlying clinical tendinopathy [1,5]. Although this model effectively reproduces innate immune activation, chronic tendinopathy involves additional mechanisms including mechanical overloading, ECM degeneration, and cellular senescence [5]. Third, the number of donor animals was limited, which may restrict the generalizability of the findings despite the use of mixed-effects models to account for inter-donor variability. Fourth, the mediator panel was restricted to selected cytokines, GF, and HA, focusing on key components of the inflammatory–regulatory–anabolic balance. However, other relevant mediators such as IL-6, IL-8, and IGF-1 were not included due to resource constraints, and future studies incorporating a broader range of analytes may provide a more comprehensive characterization of the tendon microenvironment and further elucidate the network-level dynamics underlying PRGS-mediated effects. Finally, although correlation and multivariate analyses provided insight into the organization of mediator interactions, these approaches are inherently exploratory and do not establish causal relationships among mediators. Nevertheless, the integration of mixed-effects modeling, mediator ratios, correlation network analysis, and PCA provides a comprehensive framework for examining coordinated mediator behavior in tendon inflammation and offers a systems-level perspective on the biological effects of PRG [54].

Taken together, the present findings highlight that PRGS influences the inflammatory microenvironment through coordinated and time-dependent modulation of mediator interactions. The integration of univariate, ratio-based, and multivariate analyses indicates that these effects involve simultaneous regulation of inflammatory and trophic signaling pathways, rather than isolated changes in individual mediators.

## 4. Materials and Methods

All experimental procedures were conducted in accordance with institutional and national guidelines for the care and use of animals. The study protocol was reviewed and approved by the Institutional Animal Ethics Committee on 5 September 2015 (Project Code: 0425915). Written informed consent was obtained from the owners of all donor animals prior to sample collection.

The present experiment forms part of a broader experimental program designed to investigate the biological effects of PRP-derived products in equine musculoskeletal explant systems [38,40,51,55,56]. A previous publication evaluated these biomaterials in tendon explants under non-inflammatory conditions [26]. The current study extends this work by examining tendon explants subjected to LPS-induced inflammatory stimulation.

### 4.1. Preparation and Characterization of Hemocomponents

Peripheral blood was obtained from six clinically healthy adult horses (6–10 years old; three females and three males). Whole blood was collected into 4.5 mL sodium citrate tubes (BD Vacutainer^®^, Becton Drive, Franklin Lakes, NJ, USA) and processed within an hour at room temperature using a standardized double-centrifugation tube method previously validated for equine blood [57].

Briefly, blood samples were centrifuged at 120× *g* for 5 min. Approximately 50% of the upper plasma fraction adjacent to the buffy coat was carefully aspirated under aseptic conditions and transferred to sterile tubes. This fraction underwent a second centrifugation at 240× *g* for 5 min. After this step, the lower plasma fraction was collected and designated platelet-rich plasma (PRP), while the upper fraction was considered platelet-poor plasma (PPP).

Platelet and WBC counts were determined in whole blood, PRP, and PPP using an automated hematology analyzer (Celltac-α MEK 6450, Nihon Kohden, Japan). Enrichment factors and WBC recovery were calculated to characterize the cellular profile of the hemocomponents.

To obtain supernatant fractions, both PRP and PPP were activated with calcium gluconate (1:10 *v*/*v*; Ropsohn Therapeutics Ltd.a^®^, Bogotá, Colombia) and incubated at 37 °C until clot formation and retraction occurred. The supernatant derived from activated PRP was designated PRGS, while the supernatant derived from activated PPP was designated PPGS. These fractions were used fresh for explant treatments.

PRGS and PPGS represent the soluble fractions derived from activated platelet-rich and platelet-poor plasma, respectively, and therefore differ primarily in their platelet-derived mediator content, allowing isolation of platelet-driven effects within the experimental system.

### 4.2. Tendon Procurement and Explant Preparation

SDFT samples were aseptically harvested from horses euthanized for reasons unrelated to musculoskeletal disease. Prior to tissue collection, limbs underwent clinical and ultrasonographic examination to exclude macroscopic or imaging evidence of tendinopathy.

Tendon specimens were dissected from the mid-metacarpal region and sectioned into standardized rectangular explants (approximately 5 × 3 × 3 mm; 70 ± 5 mg) under sterile conditions. Explants were rinsed in phosphate-buffered saline and stabilized for 24 h in Dulbecco’s Modified Eagle Medium (high glucose, L-glutamine supplemented) (Lonza, Basel, Switzerland) containing penicillin (100 μg/mL) and streptomycin (100 μg/mL), under serum-free conditions. Cultures were maintained at 37 °C in a humidified atmosphere with 5% CO_2_ before inflammatory stimulation.

### 4.3. Establishment of the Inflammatory Tendon Model

To generate a controlled inflammatory microenvironment, explants were stimulated with lipopolysaccharide (LPS; Sigma-Aldrich, St. Louis, MO, USA) at a final concentration of 100 ng/mL, applied as a single exposure to induce an inflammatory response. LPS was not re-added following media changes, and samples were collected at 1 h and 48 h. This concentration was selected based on prior validation studies demonstrating reproducible induction of inflammatory mediator production in equine musculoskeletal explants [38,40,51,55,56].

### 4.4. Experimental Groups and Treatment Conditions

Tendon explants were randomly allocated into six experimental conditions: non-stimulated control, LPS-stimulated control, LPS + 25% PRGS, LPS + 50% PRGS, LPS + 25% PPGS, and LPS + 50% PPGS. Supernatant fractions were added to the culture medium to achieve the indicated final concentrations. Explants were maintained under these conditions for 48 h, and culture media were collected at predefined time points for subsequent mediator quantification. The 25% concentration was selected based on previous mechanistic observations in non-inflamed tendon explants, whereas the 50% concentration was included to evaluate potential concentration-dependent effects under inflammatory challenge.

### 4.5. Quantification of Cytokines, Growth Factors, and Hyaluronic Acid

Concentrations of PDGF-BB, TGF-β1, TNF-α, IL-4, IL-1ra, IL-1β, and hyaluronic acid (HA) were determined in PRGS and PPGS immediately after preparation, as well as in explant culture supernatants collected at 1 h and 48 h following treatment.

All analytes were quantified by ELISA in duplicate using commercial development kits, according to the manufacturer’s instructions. PDGF-BB (Human PDGF-BB DuoSet, DY220, R&D Systems, Minneapolis, MN, USA) and TGF-β1 (Human TGF-β1 DuoSet, DY240E, R&D Systems, Minneapolis, MN, USA) were measured using human-specific antibodies due to the high amino acid sequence homology between human and equine proteins and documented cross-reactivity in equine biological samples [58,59]. These kits have been widely applied in equine PRP studies [60,61].

TNF-α (Equine TNF-α DuoSet, DY1814, R&D Systems, Minneapolis, MN, USA), IL-4 (Equine IL-4 DuoSet, DY1809, R&D Systems, Minneapolis, MN, USA), IL-1ra (Equine IL-1ra/IL-1F3 DuoSet, DY1814, R&D Systems, Minneapolis, MN, USA), and IL-1β (Equine IL-1β DuoSet, DY1816, R&D Systems, Minneapolis, MN, USA) were quantified using equine-specific antibodies. Hyaluronic acid was determined using a multispecies ELISA kit (Hyaluronan DuoSet, DY3614, R&D Systems, Minneapolis, MN, USA).

Standard curves were generated for each assay plate using recombinant standards supplied with the respective kits. Absorbance was measured at 450 nm with wavelength correction at 540–570 nm using a microplate reader (Multiskan MK3, Thermo Scientific, Thermo Fisher Scientific Inc., Waltham, MA, USA). Concentrations were calculated using four-parameter logistic regression.

All samples were assayed in duplicate. Intra-assay variability was monitored by calculating the coefficient of variation (CV) between replicates, and samples with CV > 10% were reanalyzed.

### 4.6. Statistical and Multivariate Analysis

All statistical analyses were performed in R (v4.5.2; R Foundation for Statistical Computing, Vienna, Austria) within a fully scripted and reproducible workflow using tidyverse (v2.0.0) for data management and visualization, lme4 (v1.1.38) and lmerTest (v3.2.1) for mixed-effects modeling, and emmeans (v2.0.1) for estimation of marginal means and post hoc contrasts [62].

Platelet and WBC counts were compared among whole blood, PRP, and PPP using linear mixed-effects models with hemocomponent as a fixed effect and horse as a random intercept to account for repeated measurements within animals [63]. Mediator concentrations measured directly in PRGS and PPGS were analyzed using paired linear models including hemocomponent as a fixed effect and horse as a blocking factor. For explant supernatant data, linear mixed-effects models were fitted for each mediator including treatment group, time, and their interaction as fixed effects, with horse identity included as a random intercept to account for clustering of multiple explants derived from the same donor. Models were estimated using restricted maximum likelihood (REML), and assumptions were assessed through inspection of residual-versus-fitted and Q–Q plots. Variance components, marginal and conditional R^2^ values, and intraclass correlation coefficients were calculated to characterize inter-horse variability and clustering effects [64].

When necessary, mediator concentrations were log10-transformed to improve normality and variance homogeneity. Estimated marginal means were obtained for all fixed-effect combinations, and pairwise contrasts were performed using Holm adjustment to control the family-wise error rate. Results were back-transformed to the original scale for interpretation. Derived mediator ratios were constructed on the log scale as differences between log-transformed variables, corresponding to log10-transformed ratios, and were expressed as fold-change ratios after back-transformation.

Pearson correlation analyses were performed at each time point using log10-transformed mediator concentrations, with false discovery rate (FDR) adjustment applied for multiple testing. Interpretation focused on moderate-to-strong associations (|r| ≥ 0.60) that remained statistically significant after FDR correction. Correlation patterns were used to identify biologically meaningful mediator modules and to guide the construction of derived ratios. Because correlation analyses describe statistical associations rather than mechanistic interactions, these results were interpreted as exploratory.

Principal component analysis (PCA) was applied to centered and scaled log-transformed mediator data, including cytokines, growth factors, hyaluronic acid, and selected derived ratios supported by the correlation structure. Stability of component loadings was assessed through bootstrap resampling at the horse level (B = 2000), and hierarchical clustering based on Euclidean distance was used to visualize mediator organization across conditions [54].

A two-sided significance threshold of *p* < 0.05 was adopted for univariate analyses. However, interpretation emphasized biological coherence, consistency of directionality, and stability of multivariate organization rather than reliance on isolated *p*-values.

## 5. Conclusions

This study provides an integrated characterization of the mediator landscape generated by PRGS in an LPS-induced tendon explant system. Rather than simply suppressing inflammatory signaling, PRGS modulated the inflammatory microenvironment through coordinated changes involving cytokines, GF, and extracellular matrix-associated mediators. Multivariate and network analyses further revealed that mediator interactions reorganized over time, shifting from an early inflammatory configuration toward a more regulated environment increasingly associated with trophic signaling and ECM-related processes.

Notably, PRGS treatment was associated with coordinated increases in regulatory mediators and with a persistent PDGF-BB–HA relationship, suggesting that PRGS-derived factors may contribute to the establishment of a balanced inflammatory–anabolic milieu compatible with tissue repair. These observations support the concept that PRGS products act as biological modulators of complex mediator networks rather than as single-target anti-inflammatory agents.

Taken together, the present findings contribute to a systems-level understanding of how PRP-related hemocomponents influence inflammatory and trophic signaling in tendon tissue. This integrated perspective may help refine the mechanistic framework underlying regenerative therapies and support the rational application of PRP-derived products in the management of musculoskeletal disorders.

## Figures and Tables

**Table 1:** Cellular composition of whole blood and derived hemocomponents (PRP and PPP).

Parameter	Whole Blood	PRP	PPP
Platelets (×10^3^/µL)	124.6 (112.3–138.3) ᵃ	308.0 (277.5–341.9) ᵇ	99.1 (89.3–110.1) ᶜ
WBC (cells/µL)	8371 (6344–11,044) ᵃ	3297 (2499–4351) ᵇ	136 (103–179) ᶜ

Values represent back-transformed estimated marginal means (EMM ± 95% CI) obtained from linear mixed-effects models including horse as a random intercept. Different superscript lowercase letters (^a–c^) indicate statistically significant differences among hemocomponents (Holm-adjusted *p* < 0.05); groups that do not share a letter differ significantly. PRP, platelet-rich plasma; PPP, platelet-poor plasma; WBC, white blood cells.

**Table 2:** Concentrations of cytokines, growth factors, and hyaluronic acid in PRGS and PPGS.

Mediator	PRGS	PPGS	t (df = 5)	*p* (Holm)
TGF-β1 (pg/mL)	2661 (1783–3539)	1263 (385–2141)	2.89	0.034
PDGF-BB (pg/mL)	2044 (1382–2706)	261 (−401–922)	4.90	0.004
TNF-α (pg/mL)	61.6 (60.5–62.7)	58.9 (57.8–60.1)	4.34	0.007
IL-4 (pg/mL)	66.9 (57.7–76.0)	55.4 (46.2–64.6)	2.27	0.072
IL-1ra (pg/mL)	1196 (424–1967)	66.9 (−705–838)	2.66	0.045
HA (ng/mL)	1.03 (0.65–1.41)	0.99 (0.61–1.37)	0.21	0.845

Values represent back-transformed estimated marginal means (EMM ± 95% CI) obtained from paired linear models including horse as a blocking factor. Holm-adjusted contrasts were used for pairwise comparisons, and statistical differences are indicated by the corresponding *p*-values. IL-1β concentrations in PRGS and PPGS supernatants were not reported quantitatively because values were outside the validated quantification conditions of the assay for these supernatants. PRGS, platelet-rich gel supernatant; PPGS, platelet-poor gel supernatant; PDGF-BB, platelet-derived growth factor-BB; TGF-β1, transforming growth factor beta-1; TNF-α, tumor necrosis factor alpha; IL-4, interleukin-4; IL-1ra, interleukin-1 receptor antagonist; HA, hyaluronic acid.

**Table 3:** Type III ANOVA results from linear mixed-effects models for individual mediators in the LPS-induced tendon explant system.

Mediator	Effect	NumDF	DenDF	F	*p* Value
TGF-β1	Group	5	55	0.387	0.856
	Time	1	55	0.903	0.346
	Group × Time	5	55	1.378	0.247
PDGF-BB	Group	5	60	48.145	<0.001
	Time	1	60	0.385	0.537
	Group × Time	5	60	0.616	0.688
TNF-α	Group	5	55	1.602	0.175
	Time	1	55	0.201	0.655
	Group × Time	5	55	2.218	0.065
IL-4	Group	5	60	1.142	0.348
	Time	1	60	0.213	0.646
	Group × Time	5	60	13.610	<0.001
IL-1ra	Group	5	55	38.593	<0.001
	Time	1	55	0.500	0.482
	Group × Time	5	55	0.771	0.575
IL-1β	Group	5	55	0.739	0.598
	Time	1	55	8.675	0.005
	Group × Time	5	55	13.317	<0.001
HA	Group	5	55	0.449	0.812
	Time	1	55	10.752	0.002
	Group × Time	5	55	5.815	<0.001

Linear mixed-effects models included treatment group, time, and their interaction as fixed effects, with horse identity as a random intercept. Type III ANOVA tests were performed on log10-transformed data using restricted maximum likelihood estimation. NumDF, numerator degrees of freedom; DenDF, denominator degrees of freedom. Other abbreviations as in Table 2.

**Table 4:** Type III ANOVA results from linear mixed-effects models for inflammatory–matrix ratios in the LPS-induced tendon explant system.

Ratio	Effect	NumDF	DenDF	F	*p* Value
IL-1β:HA	Group	5	55	1.349	0.258
	Time	1	55	3.214	0.079
	Group × Time	5	55	10.499	<0.001
IL-1β:IL-4	Group	5	55	1.408	0.236
	Time	1	55	1.972	0.166
	Group × Time	5	55	3.651	0.006
PDGF-BB:HA	Group	5	55	46.100	<0.001
	Time	1	55	0.218	0.643
	Group × Time	5	55	0.705	0.622

Linear mixed-effects models included treatment group, time, and their interaction as fixed effects, with horse identity as a random intercept. Type III ANOVA tests were performed on log10-transformed ratios using restricted maximum likelihood estimation. Abbreviations as in Table 2 and Table 3.

**Table 5:** Type III ANOVA results from linear mixed-effects models fitted to PC1 and PC2 scores.

Response	Effect	NumDF	DenDF	F	*p* Value
PC1	Group	5	55	1.035	0.406
	Time	1	55	6.790	0.012
	Group × Time	5	55	14.602	<0.001
PC2	Group	5	55	62.542	<0.001
	Time	1	55	0.806	0.373
	Group × Time	5	55	1.487	0.209

Linear mixed-effects models included treatment group, time, and their interaction as fixed effects, with horse identity as a random intercept. Type III ANOVA tests were performed on PC scores derived from the principal component analysis. PC, principal component. Abbreviations as in Table 2.

**Table 6:** Bootstrap stability of PCA loadings.

Mediator	PC1 Loading (Median)	95% CI	PC2 Loading (Median)	95% CI
IL-1β	−0.556	−0.590 to −0.506	−0.054	−0.269 to 0.243
IL-1ra	−0.125	−0.340 to 0.233	0.651	0.537 to 0.700
IL-4	−0.456	−0.517 to −0.330	−0.131	−0.453 to 0.162
TNF-α	−0.335	−0.446 to −0.177	0.095	−0.270 to 0.352
PDGF-BB	−0.109	−0.350 to 0.225	0.653	0.534 to 0.699
TGF-β1	0.283	0.078 to 0.414	0.173	−0.205 to 0.439
HA	−0.478	−0.536 to −0.390	−0.083	−0.303 to 0.196

Bootstrap resampling (2000 iterations) was used to estimate the stability of mediator loadings on the first two principal components. Values represent the median loading and the 95% bootstrap confidence interval.

## Data Availability

The raw data supporting the conclusions of this article will be made available by the authors upon request.

## Abbreviations

The following abbreviations are used in this manuscript:

CI	Confidence interval
ECM	Extracellular matrix
EMM	Estimated marginal mean
FDR	False discovery rate
GF	Growth factors
HA	Hyaluronic acid
IL-1β	Interleukin-1 beta
IL-1ra	Interleukin-1 receptor antagonist
IL-4	Interleukin-4
LPS	Lipopolysaccharide
PCA	Principal component analysis
PDGF-BB	Platelet-derived growth factor-BB
PPGS	Platelet-poor gel supernatant
PPP	Platelet-poor plasma
PRG	Platelet-rich gel
PRGS	Platelet-rich gel supernatant
PRP	Platelet-rich plasma
SDFT	Superficial digital flexor tendon
TGF-β1	Transforming growth factor beta 1
TLR4	Toll-like receptor 4
TNF-α	Tumor necrosis factor alpha
WBC	White blood cells

## References

[1] Lui P.P.Y., Maffulli N., Rolf C., Smith R.K.W. (2011). What Are the Validated Animal Models for Tendinopathy?. Scand. J. Med. Sci. Sports.

[2] Oreff G.L., Fenu M., Vogl C., Ribitsch I., Jenner F. (2021). Species Variations in Tenocytes’ Response to Inflammation Require Careful Selection of Animal Models for Tendon Research. Sci. Rep..

[3] Clegg P.D. (2012). Musculoskeletal Disease and Injury, Now and in the Future. Part 2: Tendon and Ligament Injuries. Equine Vet. J..

[4] Smith R.K.W., McIlwraith C.W. (2021). “One Health” in Tendinopathy Research: Current Concepts. J. Orthop. Res..

[5] Sharma P., Maffulli N. (2005). Tendon Injury and Tendinopathy: Healing and Repair. J. Bone Jt. Surg. Am..

[6] Dziekoński K., Popiel M., Wieczorek I., Cybulski P., Gorycki H., Wacławek W., Matwiejuk W., Samek J., Komorowski-Roszkiewicz J., Marcyś K. (2025). Achilles Tendinopathy: Epidemiology, Diagnosis, and Treatment Strategies—A Review. Qual. Sport.

[7] Millar N.L., Murrell G.A., McInnes I.B. (2017). Inflammatory Mechanisms in Tendinopathy–towards Translation. Nat. Rev. Rheumatol..

[8] Mosca M.J., Rashid M.S., Snelling S.J., Kirtley S., Carr A.J., Dakin S.G. (2018). Trends in the Theory That Inflammation Plays a Causal Role in Tendinopathy: A Systematic Review and Quantitative Analysis of Published Reviews. BMJ Open Sport Exerc. Med..

[9] Jun T., Ruipeng G., Bin X. (2020). TLR4 Knockdown by miRNA-140-5p Improves Tendinopathy: An in Vitro Study. Arch. Med. Sci. AMS.

[10] Jiang L., Liu T., Lyu K., Chen Y., Lu J., Wang X., Long L., Li S. (2023). Inflammation-Related Signaling Pathways in Tendinopathy. Open Life Sci..

[11] Dakin S.G., Martinez F.O., Yapp C., Wells G., Oppermann U., Dean B.J., Smith R.D., Wheway K., Watkins B., Roche L. (2015). Inflammation Activation and Resolution in Human Tendon Disease. Sci. Transl. Med..

[12] Manning C.N., Havlioglu N., Knutsen E., Sakiyama-Elbert S.E., Silva M.J., Thomopoulos S., Gelberman R.H. (2014). The Early Inflammatory Response after Flexor Tendon Healing: A Gene Expression and Histological Analysis. J. Orthop. Res..

[13] Li H., Luo S., Wang H., Chen Y., Ding M., Lu J., Jiang L., Lyu K., Huang S., Shi H. (2023). The Mechanisms and Functions of TGF-Β1 in Tendon Healing. Injury.

[14] Kovacevic D., Gulotta L.V., Ying L., Ehteshami J.R., Deng X.-H., Rodeo S.A. (2015). rhPDGF-BB Promotes Early Healing in a Rat Rotator Cuff Repair Model. Clin. Orthop. Relat. Res..

[15] Yagishita K., Sekiya I., Sakaguchi Y., Shinomiya K., Muneta T. (2005). The Effect of Hyaluronan on Tendon Healing in Rabbits. Arthroscopy.

[16] Kaux J.-F., Samson A., Crielaard J.-M. (2016). Hyaluronic Acid and Tendon Lesions. Muscles Ligaments Tendons J..

[17] Bahadir B., Sarikaya B. (2024). Platelet-Rich Plasma in the Management of Rotator Cuff Tendinopathy. Jt. Dis. Relat. Surg..

[18] de Vos R.J., Weir A., van Schie H.T.M., Bierma-Zeinstra S.M.A., Verhaar J.A.N., Weinans H., Tol J.L. (2010). Platelet-Rich Plasma Injection for Chronic Achilles Tendinopathy A Randomized Controlled Trial. JAMA-J. Am. Med. Assoc..

[19] Fortier L.A., Smith R.K. (2008). Regenerative Medicine for Tendinous and Ligamentous Injuries of Sport Horses. Vet. Clin. N. Am. Equine Pract..

[20] Marx R.E. (2001). Platelet-Rich Plasma (PRP): What Is PRP and What Is Not PRP?. Implant Dent..

[21] Anitua E., Andia I., Ardanza B., Nurden P., Nurden A.T. (2004). Autologous Platelets as a Source of Proteins for Healing and Tissue Regeneration. Thromb. Haemost..

[22] Dohan Ehrenfest D.M., Andia I., Zumstein M.A., Zhang C.Q., Pinto N.R., Bielecki T. (2014). Classification of Platelet Concentrates (Platelet-Rich Plasma-PRP, Platelet-Rich Fibrin-PRF) for Topical and Infiltrative Use in Orthopedic and Sports Medicine: Current Consensus, Clinical Implications and Perspectives. Muscles Ligaments Tendons J..

[23] Camargo Garbin L., Lopez C., Carmona J.U. (2021). A Critical Overview of the Use of Platelet-Rich Plasma in Equine Medicine Over the Last Decade. Front. Vet. Sci..

[24] Escobar G., Escobar A., Ascui G., Tempio F.I., Ortiz M.C., Pérez C.A., López M.N. (2018). Pure Platelet-Rich Plasma and Supernatant of Calcium-Activated P-PRP Induce Different Phenotypes of Human Macrophages. Regen. Med..

[25] Xiao S., Wang J., Chen Q., Miao Y., Hu Z. (2019). The Mechanism of Activated Platelet-rich Plasma Supernatant Promotion of Hair Growth by Cultured Dermal Papilla Cells. J. Cosmet. Dermatol..

[26] Bonilla-Gutiérrez A.F., Castillo-Franz C., López C., Álvarez M.E., Giraldo C.E., Carmona J.U. (2018). Equine Suspensory Ligament and Tendon Explants Cultured with Platelet-Rich Gel Supernatants Release Different Anti-Inflammatory and Anabolic Mediators. Biomed. Pharmacother..

[27] Kitano H. (2002). Systems Biology: A Brief Overview. Science.

[28] Vodovotz Y. (2010). Translational Systems Biology of Inflammation and Healing. Wound Repair Regen..

[29] Vodovotz Y., An G. (2010). Systems Biology and Inflammation. Methods Mol. Biol..

[30] Berkoff D.J., Kallianos S.A., Eskildsen S.M., Weinhold P.S. (2016). Use of an IL1-receptor Antagonist to Prevent the Progression of Tendinopathy in a Rat Model. J. Orthop. Res..

[31] Eskildsen S.M., Berkoff D.J., Kallianos S.A., Weinhold P.S. (2019). The Use of an IL1-receptor Antagonist to Reverse the Changes Associated with Established Tendinopathy in a Rat Model. Scand. J. Med. Sci. Sports.

[32] Haupt J.L., Donnelly B.P., Nixon A.J. (2006). Effects of Platelet-Derived Growth Factor-BB on the Metabolic Function and Morphologic Features of Equine Tendon in Explant Culture. Am. J. Vet. Res..

[33] Cooke J.P. (2019). Inflammation and Its Role in Regeneration and Repair: A Caution for Novel Anti-Inflammatory Therapies. Circ. Res..

[34] Favier A.-L., Nikovics K. (2023). Molecular and Cellular Mechanisms of Inflammation and Tissue Regeneration. Biomedicines.

[35] Liu Y., Wang L., Li S., Zhang T., Chen C., Hu J., Sun D., Lu H. (2022). Mechanical Stimulation Improves Rotator Cuff Tendon-Bone Healing via Activating IL-4/JAK/STAT Signaling Pathway Mediated Macrophage M2 Polarization. J. Orthop. Transl..

[36] Courneya J.-P., Luzina I.G., Zeller C.B., Rasmussen J.F., Bocharov A., Schon L.C., Atamas S.P. (2010). Interleukins 4 and 13 Modulate Gene Expression and Promote Proliferation of Primary Human Tenocytes. Fibrogenesis Tissue Repair.

[37] Morita W., Dakin S.G., Snelling S.J.B., Carr A.J. (2017). Cytokines in Tendon Disease: A Systematic Review. Bone Jt. Res..

[38] Carmona J.U., Ríos D.L., López C., Álvarez M.E., Pérez J.E., Bohórquez M.E. (2016). In Vitro Effects of Platelet-Rich Gel Supernatants on Histology and Chondrocyte Apoptosis Scores, Hyaluronan Release and Gene Expression of Equine Cartilage Explants Challenged with Lipopolysaccharide. BMC Vet. Res..

[39] Li H., Li Y., Luo S., Zhang Y., Feng Z., Li S. (2024). The Roles and Mechanisms of the NF-κB Signaling Pathway in Tendon Disorders. Front. Vet. Sci..

[40] Castillo-Franz C., López C., Carmona J.U. (2021). Evaluation of the Catabolic and Anabolic Gene Expression Effects and Histology Changes Induced by Platelet-Rich Gel Supernatants in Equine Suspensory Ligament Explants Challenged with Lipopolysaccharide. MLTJ-Muscles Ligaments Tendons J..

[41] Boswell S.G., Schnabel L.V., Mohammed H.O., Sundman E.A., Minas T., Fortier L.A. (2014). Increasing Platelet Concentrations in Leukocyte-Reduced Platelet-Rich Plasma Decrease Collagen Gene Synthesis in Tendons. Am. J. Sports Med..

[42] Cengiz I.F., Oliveira J.M., Reis R.L. (2018). PRP Therapy. Adv. Exp. Med. Biol..

[43] Greenspoon J.A., Moulton S.G., Millett P.J., Petri M. (2016). The Role of Platelet Rich Plasma (PRP) and Other Biologics for Rotator Cuff Repair. Open Orthop. J..

[44] Schnabel L.V., Mohammed H.O., Miller B.J., McDermott W.G., Jacobson M.S., Santangelo K.S., Fortier L.A. (2007). Platelet Rich Plasma (PRP) Enhances Anabolic Gene Expression Patterns in Flexor Digitorum Superficialis Tendons. J. Orthop. Res..

[45] Baldo B.A. (2014). Side Effects of Cytokines Approved for Therapy. Drug Saf..

[46] Lin J., Ziring D., Desai S., Kim S., Wong M., Korin Y., Braun J., Reed E., Gjertson D., Singh R.R. (2008). TNFα Blockade in Human Diseases: An Overview of Efficacy and Safety. Clin. Immunol..

[47] van Dissel J.T., Van Langevelde P., Westendorp R.G., Kwappenberg K., Frölich M. (1998). Anti-Inflammatory Cytokine Profile and Mortality in Febrile Patients. Lancet.

[48] Dinarello C.A. (2000). Proinflammatory Cytokines. Chest.

[49] Efron B. (1987). Better Bootstrap Confidence Intervals. J. Am. Stat. Assoc..

[50] Efron B., Tibshirani R.J. (1994). An Introduction to the Bootstrap.

[51] Ríos D.L., López C., Álvarez M.E., Samudio I.J., Carmona J.U. (2015). Effects over Time of Two Platelet Gel Supernatants on Growth Factor, Cytokine and Hyaluronan Concentrations in Normal Synovial Membrane Explants Challenged with Lipopolysaccharide. BMC Musculoskelet. Disord..

[52] Wunderli S.L., Blache U., Snedeker J.G. (2020). Tendon Explant Models for Physiologically Relevant in Vitro Study of Tissue Biology–a Perspective. Connect. Tissue Res..

[53] Szczesny S.E., Corr D.T. (2023). Tendon Cell and Tissue Culture: Perspectives and Recommendations. J. Orthop. Res..

[54] Jolliffe I.T., Cadima J. (2016). Principal Component Analysis: A Review and Recent Developments. Philos. Trans. A Math. Phys. Eng. Sci..

[55] Ríos D.L., López C., Carmona J.U. (2015). Evaluation of the Anti-Inflammatory Effects of Two Platelet-Rich Gel Supernatants in an in Vitro System of Cartilage Inflammation. Cytokine.

[56] Castillo-Franz C., López C., Álvarez M.E., Giraldo C.E., Carmona J.U. (2019). Anti-Inflammatory Effects of Two Platelet-Rich Gel Supernatants in an in Vitro System of Ligament Desmitis. Muscles Ligaments Tendons J..

[57] Carmona J.U., López C., Jurado-Grisales C. (2025). A Simple Double Centrifugation Tube Method to Obtain Platelet-Rich Plasma from Equine Blood. J. Vis. Exp..

[58] Donnelly B.P., Nixon A.J., Haupt J.L., Dahlgren L.A. (2006). Nucleotide Structure of Equine Platelet-Derived Growth Factor-A and-B and Expression in Horses with Induced Acute Tendinitis. Am. J. Vet. Res..

[59] Penha-goncalves M.N., Onions D.E., Nicolson L. (1997). Cloning and Sequencing of Equine Transforming Growth Factor-Beta 1 (TGFβ-1) cDNA. DNA Seq..

[60] Giraldo C.E., Álvarez M.E., Carmona J.U. (2015). Effects of Sodium Citrate and Acid Citrate Dextrose Solutions on Cell Counts and Growth Factor Release from Equine Pure-Platelet Rich Plasma and Pure-Platelet Rich Gel. BMC Vet. Res..

[61] Giraldo C.E., López C., Álvarez M.E., Samudio I.J., Prades M., Carmona J.U. (2013). Effects of the Breed, Sex and Age on Cellular Content and Growth Factor Release from Equine Pure-Platelet Rich Plasma and Pure-Platelet Rich Gel. BMC Vet. Res..

[62] Team R.C. (2016). R: A Language and Environment for Statistical Computing.

[63] Pinheiro J.C., Bates D.M. (2000). Mixed-Effects Models in S and S-PLUS.

[64] Zuur A.F., Ieno E.N., Walker N.J., Saveliev A.A., Smith G.M. (2009). Mixed Effects Models and Extensions in Ecology with R.

